# A Narrow-Band Multi-Resonant Metamaterial in Near-IR

**DOI:** 10.3390/ma13225140

**Published:** 2020-11-14

**Authors:** Farhan Ali, Serap Aksu

**Affiliations:** 1 Department of Physics, Bilkent University, Ankara 06800, Turkey; fali19@ku.edu.tr; 2 Department of Physics, Koc University, Istanbul 34450, Turkey

**Keywords:** multi-resonant nanoantennas, narrow-band perfect absorbers, NIR spectroscopy, critical coupling, impedance matching, contrast agent for imaging

## Abstract

We theoretically investigate a multi-resonant plasmonic metamaterial perfect absorber operating between 600 and 950 nm wavelengths. The presented device generates 100% absorption at two resonance wavelengths and delivers an ultra-narrow band (sub-20 nm) and high quality factor (Q=44) resonance. The studied perfect absorber is a metal–insulator–metal configuration where a thin MgF${}_{2}$ spacer is sandwiched between an optically thick gold layer and uniformly patterned gold circular nanodisc antennas. The localized and propagating nature of the plasmonic resonances are characterized and confirmed theoretically. The origin of the perfect absorption is investigated using the impedance matching and critical coupling phenomenon. We calculate the effective impedance of the perfect absorber and confirm the matching with the free space impedance. We also investigate the scattering properties of the top antenna layer and confirm the minimized reflection at resonance wavelengths by calculating the absorption and scattering cross sections. The excitation of plasmonic resonances boost the near-field intensity by three orders of magnitude which enhances the interaction between the metamaterial surface and the incident energy. The refractive index sensitivity of the perfect absorber could go as high as S=500 nm/RIU. The presented optical characteristics make the proposed narrow-band multi-resonant perfect absorber a favorable platform for biosensing and contrast agent based bioimaging.

## 1. Introduction

Plasmonic metamaterial perfect absorbers (PA) [1,2,3,4,5,6] have attracted tremendous attention as they can fully absorb the incident light at optical frequencies [7,8,9,10,11,12] and allow a variety of exotic applications [13,14,15,16,17]. PAs can be typically designed using three functional layers that comprise a dielectric interlayer sandwiched between the bottom metal film and a patterned top metal plasmonic nanoantenna serving as a resonator [18,19,20,21,22]. The three-layered configuration includes an important parameter, dielectric layer thickness, that can bring the absorption level to unity (100%) and width of the resonance (full width at half-maximum (FWHM)) below 20 nm in wavelength. PAs are generally categorized with respect to their resonance bandwidths and operational frequency. In the literature, broadband resonant PAs are investigated in more detail when compared to narrowband PAs, as their large absorption bandwidth in the near-infrared (NIR) region (600–950 nm) makes them an ideal candidate for energy harvesting [12,23]. On the other hand, narrowband PAs with high quality-factor resonances has been an ideal solution for biosensing and bioimaging applications [5,15,24,25,26]. In particular, PAs providing a multi-band and narrow resonance behavior in the NIR region can potentially work as efficient targeted contrast agents for photoacoustic medical imaging tools, and facilitate selective imaging of low concentration biomolecules in deep tissue [27,28,29]. However, unlike mid-infrared frequencies, the study of multi-resonant narrow-band perfect absorbers for NIR has been limited. Previously suggested narrowband PAs for NIR are single-resonant and the discussion of the physical origin of the perfect absorption is generally omitted [22,24,25,30].

In this work, we present a multi-resonant plasmonic metamaterial perfect absorber operating in the NIR region. The presented PA shows near-unity absorption at 653 nm and 865 nm wavelengths and delivers an ultra-narrow band (sub 20 nm) and high quality factor (Q=44) resonance. The PA is composed of a multilayer metal–insulator–metal (MIM) configuration where a thin (40 nm) MgF${}_{2}$ spacer is sandwiched between an optically thick (100 nm) gold bottom layer and uniformly patterned gold circular nanodiscs on the top layer. The nanodisc antenna-patterned MIM configuration as a single resonant metamaterial has been studied before in the infrared region [26]. We present a delicate control over structural parameters that enable the multi-resonant behavior in the NIR. Theoretical studies revealed that the origins of the two absorption peaks are different, one arises from the excitation of propagating surface plasmons (PSPs), whereas the other is due to the excitation of localized surface plasmons (LSP). Upon exposure to the incident light, our theoretical study confirmed that effective impedance of the multilayer system matches the free space impedance. In addition, the radiative and intrinsic damping rates of the system becomes equal at the resonance frequencies resulting in identical absorption and scattering cross sections. At the resonant wavelengths the excitation of the plasmonic resonances boost the electromagnetic field intensity around the nanodiscs ~$10^{3}$-fold. Calculations showed that refractive index sensitivity of the perfect absorber could go as high as S=500 nm/RIU. Its dual resonant characteristic with 100% absorption, high Q-factor resonances, and near-field intensity peaking at the resonant frequencies make the proposed narrow resonance perfect absorbers a favorable platform for biosensing and bioimaging applications at various wavelengths.

We study a multilayer metal–insulator–metal (MIM) configuration composed of MgF${}_{2}$ spacer, gold bottom layer, and uniformly patterned gold circular nanodiscs on the top layer. The structural analysis shows that the studied PA can exhibit higher absorption intensity when a dielectric spacer with finite thickness is sandwiched between ground metal plate and top metal antenna layer as opposed to double layer of metal–dielectric or metal–metal (Figure S3). In addition, changing the shape of the top antenna layer do not dramatically reduce the absorption for the same antenna size. Therefore, with the tuning of the antenna dimension near-unity absorption and multi-resonant behavior could be achieved with a variety of different antenna shapes (Figure S5) in NIR. As the antenna shape is not critical to achieve near-unity absorption, we have chosen to study a basic shape, a disc, as it is easier to fabricate when compared to other structures with corners. Moreover, the disc shape is insensitive to polarization of the incident light (Figure S1). The effect of antenna size and thicknesses are discussed in detail in Structure Design and Numerical Analysis part. In the context of chemical-physical analysis, gold is chosen as metal layers due to its resistance to oxidation. The effect of different dielectric materials for perfect absorption is discussed in the Figure S4. The change of refractive index in the range of 1 to 1.7 does not dramatically reduce the absorption. Hence, with the tuning of the dielectric layer thickness near-unity absorption and multi-resonant behavior could be achieved with a variety of different materials, thus with a variety of different refractive indices. We chose to study MgF${}_{2}$ due to its high optical quality in NIR and its availability in cleanrooms for fabrication.

Considering that the incident light can be reflected, transmitted, or absorbed through the PA, maximum absorption (*A*) is realized by minimizing the reflection (*R*) and transmission (*T*) as R+T+A=1. In order to achieve the perfect absorption (A≈1) with the suggested PA, transmission is minimized by an optically thick gold layer on the bottom that does not allow any incident light to pass (T≈0). Antenna theory states that zero reflection can be achieved by critically tuning the dielectric thickness layer and its optical properties (permittivity and permeability) such that the reflected light is totally suppressed via the impedance matching [31,32,33]. On the other hand, Alaee et al. presents the global limitations for achieving maximum absorption with metamaterials composed of nanoantennas [34]. To control the absorption properties, it is crucial to study the scattering properties of the individual nanodiscs which are operating as nanoantennas. A nanoantenna which exhibits only an electric dipole response is most absorptive if the absorbed power is identical to its scattered power. This condition is known as critical coupling [35,36,37,38] and appears when the radiative and intrinsic (non-radiative) damping rates, $\gamma_{r}$ and $\gamma_{i}$, respectively, of the designed system becomes identical to each other via precise selection of the dielectric spacer thickness. This leads to similar absorption and scattering cross sections ($C_{\mathrm{abs}}\approx C_{\mathrm{sca}}$ ), and most of the incident energy is absorbed by the system [34,39]. In this work, we present the underlying physics of perfect absorption at nanodisc antenna patterned MIM design from two aspects. First, we quantitatively present the impedance and optical parameters of the PA system and confirm the impedance matching of the system with air. Second, we quantify the absorption and scattering cross section of a single nanodisc antenna and confirm the critical coupling condition. Both mechanisms result in zero reflection.

## 2. Structure Design and Numerical Analysis

To calculate the near- and far-field optical properties, the proposed PA unit cell is simulated using commercially available software (Lumerical Inc., Vancouver, BC, Canada) [40]. The finite difference time domain (FDTD) method is employed for calculations. We vary the PA parameters (geometric dimensions) in a wide range to obtain the values that lead to perfect absorption. We then numerically investigate the physical mechanism of the perfect absorption and its relation with the geometric dimension. The proposed structure is studied within the wavelength range of 400 to 1000 nm. The optical constants of gold and glass are taken from Palik and the refractive index of MgF${}_{2}$ is set to n=1.377 [41,42]. As the PA has a periodic structure, instead of simulating the whole structure, only a single unit cell can be simulated by applying proper boundary conditions in all directions. Here, a single unit cell is used to simulate the reflection spectrum, and a TM polarized plane wave is incident normally from the top along the *z*-axis. Periodic boundary conditions are imposed along both *x* and *y* directions, while perfectly matched layers (PML) are used along the propagation direction (*z*-axis).

**Figure 1:**
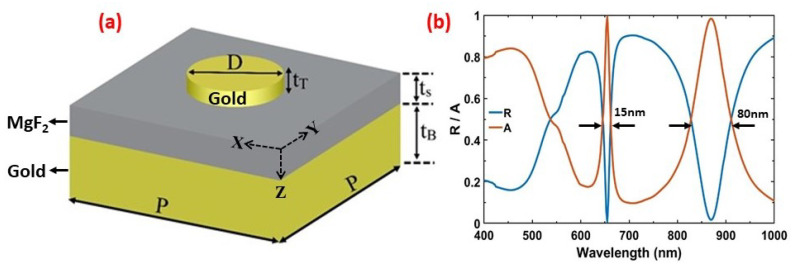
(**a**) A schematic of the unit cell of the proposed MIM metamaterial absorber with periodicity *P*. The optimized structural parameters are P=600 nm, $t_{B}=100$ nm, $t_{S}$ = $t_{T}=40$ nm, and D=180 nm. (**b**) Simulated reflection and absorption spectrum of the optimized metamaterial, yielding perfect absorption at dual spectral band at 653 nm and 865 nm with FWHM of 15 nm and 80 nm, respectively.

A schematic diagram of the unit cell and its structural parameters are shown in Figure 1a. The MIM structure consists of a top gold nanodisc antenna with thickness $t_{T}$ and diameter *D*. The middle layer of MgF${}_{2}$ with a thickness of $t_{S}$ separates the nanodisc from the bottom gold film of thickness $t_{B}$. The unit cell repeats itself in the *x* and *y* directions with a period *P*, resulting in the formation of an array. The whole system sits on a glass substrate.

A variety of simulations are performed to optimize the geometrical dimension of the system delivering the maximum absorption. The thickness of the bottom gold film is set at 100 nm, which is greater than the penetration depth of electromagnetic waves in this regime, thus suppressing any incident light transmitted through the two upper layers and ensuring the transmission to be zero, whereas the MgF${}_{2}$ spacer and nanodiscs have the same thickness of 40 nm with an array period 600 nm. Figure 1b shows the calculated reflection/absorption spectra for the nanodiscs with the mentioned optimized parameters. The absorption of the system is calculated using the relation A=1−R. Two absorption peaks can be seen at resonant wavelengths around 653 nm and 865 nm, with 99% absorption. The first resonant peak (Peak1) is narrower with a FWHM of 15 nm, whereas the second resonance peak (Peak2) has wider bandwidth with FWHM≈80 nm due to higher radiative loses. The peak excited at 653 nm appears due to the periodic gold nanodiscs array, which delivers an additional momentum $G=(2\pi /P\sqrt{m^{2}+n^{2}})$, where *P* is the periodicity and (m,n) are the grating orders of the array along both lateral directions, thus coupling the incident light into PSPs on the gold layer. It corresponds to the PSP (m=1,n=0) mode on the gold film at the gold/air interface according to the following equation,

(1) $$\lambda_{\mathrm{PSP}}=\frac{P}{\sqrt{m^{2}+n^{2}}}\sqrt{\frac{\epsilon_{m}\epsilon_{d}}{\epsilon_{m}+\epsilon_{d}}}$$

where $\epsilon_{m}$ and $\epsilon_{d}$ are the permittivity of the metal and the dielectric layer, respectively [43]. The calculated resonance wavelength for Peak1 at (1,0) mode is 639 nm, which is very close to the simulated resonance wavelength (653 nm). The excitation wavelength of PSPs strongly depends on the refractive indices of gold and the surrounding material. Meanwhile, the peak at 865 nm is in well agreement with the resonance excited in the case of a single nanodisc, thus corresponds to the LSP resonance of the top gold nanodisc. The relevant resonant frequency is determined by the geometry (size and shape) of the top resonating nanodisc as well as the surrounding material’s dielectric constant [43]. The effect of the shape of top resonating nanoantenna is discussed in Figure S5 in detail. Furthermore, we note that the structure under study is polarization independent due to the circular symmetry of the top nanodisc array, which responds similarly to the incident light for different polarization states (Figure S1) [44]. Moreover, we study the dependence on the angle of the incident light. The angle changes up to 10${}^{\circ }$ does not significantly affect the quality of the narrowband perfect absorption (Figure S2).

**Figure 2:**
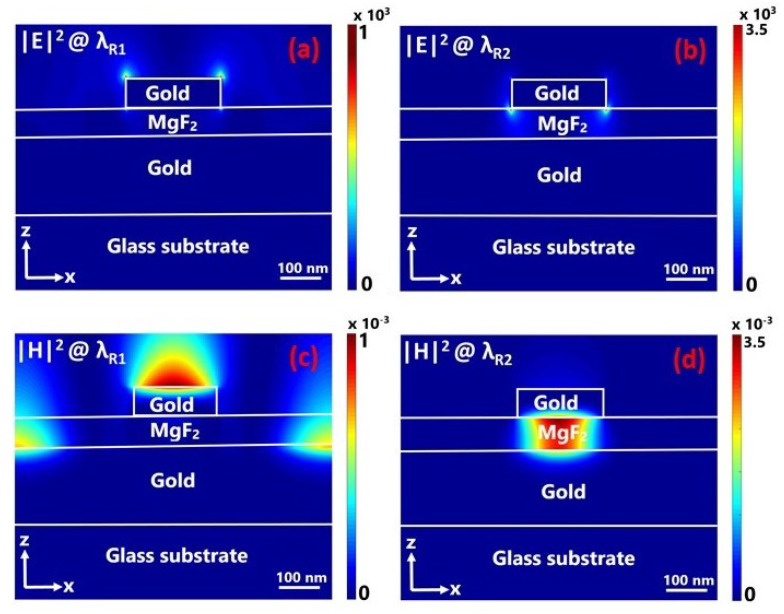
Cross-sectional view of the simulated electric field intensity ${\left|E\right|}^{2}$ and magnetic field intensity ${\left|H\right|}^{2}$ at resonant wavelengths of $\lambda_{R1}=653$ nm (**a**,**c**) and $\lambda_{R2}=865$ nm (**b**,**d**), respectively. Electric field intensity at both resonant wavelengths is enhanced by a factor of up to $10^{3}$, around the edges of the nanodisc. This confirms that the first resonant mode is excited with incident light coupling into the propagating surface plasmons (PSPs) on the gold film. On the other hand the second resonant mode is a fundamental LSP.

We can also characterize the PSP and LSP behavior of the resonances using the near-field characteristics of the metamaterial. Figure 2 shows the calculated electric and magnetic field intensities at the resonance wavelengths. The field distributions are calculated at y=0 along the xz cross section using the frequency-domain field profile monitor in the Lumerical FDTD solver for the *x*-polarized plane wave. As shown in Figure 2a, the electric field intensity at $\lambda_{R1}\approx 653$ nm is mostly confined along the boundaries of the gold nanodisc with up to one-thousand-fold field enhancement around the top edges of the gold nanodisc. On the other hand for the resonant mode at $\lambda_{R2}\approx$ 865 nm the field is mostly concentrated around the lower edges of the nanodisc, along the MgF${}_{2}$ interface and in the same order of magnitude with the first mode. In contrast to the electric field distributions, magnetic field distribution profiles presented distinct characteristics for both resonant modes as shown in Figure 2c,d. In the first resonant mode, the magnetic field is mostly confined along the nanodisc/air boundary and between the discs in the dielectric spacer/bottom gold film interface as shown in Figure 2c. Meanwhile, Figure 2d shows that for the second resonance mode the magnetic field is concentrated below the nanodisc within the dielectric. This confirms that the first resonant mode appears due to the incident light coupling into the PSPs on the gold film. On the other hand, the second resonant mode is a fundamental LSP resonance since the magnetic field is mostly confined and localized in the middle dielectric spacer region presenting no efficient coupling between neighboring nanodiscs.

**Figure 3:**
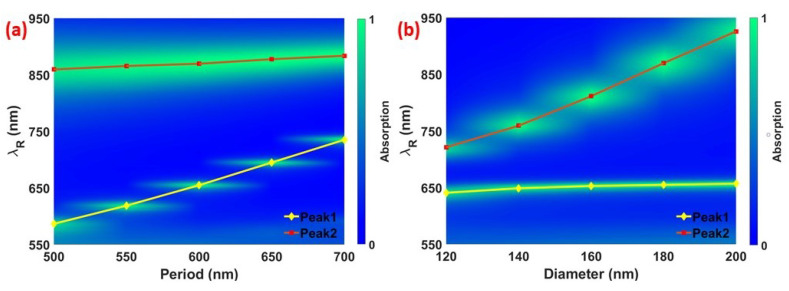
Absorption and resonant wavelength $\lambda_{R}$ of the system under study as a function of the array periodicity *P* (**a**) and diameter of the gold nanodisc *D* (**b**). The change on *P* and *D* present no significant effect on absorption. The dependence of Peak1 on the period confirms its PSP behavior, whereas dependence of Peak2 on the diameter confirms its LSP behavior.

To further confirm the physical origin of these absorption peaks and performance of the proposed structure, we investigated the influence of gold nanodisc’s diameter and array’s periodicity on the absorption peaks’ wavelength and efficiency. All the other geometrical dimensions are kept the same unless mentioned otherwise. Figure 3 shows the resonant wavelength and absorption amplitude of the proposed structure as a function of array periodicity *P* and the diameter *D* of the gold nanodiscs. Figure 3a shows that as the period of array altered from 500 to 700 nm with 50 nm steps, Peak1 at the first resonant mode shows a red shift; however, there is no significant change on the resonance wavelength for Peak2 at the second resonant mode. This data confirms that Peak1 appears due to the excitation of propagating surface plasmons on the gold film as it is strongly dependent on the period, and consequently on the array. In addition, we calculate the resonance wavelength using equation 1 as a function of P and the calculated resonance wavelengths closely match with the simulated ones. Figure 3b shows that as the diameter of the array is altered from 120 to 200 nm with 20 nm steps, Peak2 at the second resonant mode shows a red shift and there is no change on the resonance wavelength of Peak1. Changing *D* causes a shift in the fundamental LSP resonance of the individual antennas without effecting the PSP resonance. This result also confirms the localized nature of Peak2. Moreover, the change on *P* and *D* present no significant effect on the absorption amplitude of Peak1 or Peak2.

**Figure 4:**
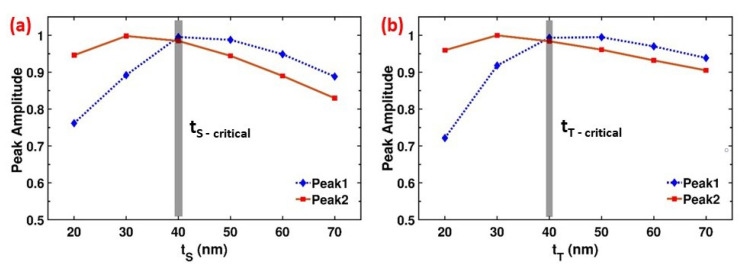
Dependence of the absorption peak amplitude on dielectric spacer thickness $t_{S}$ (**a**) and top nanodisc thickness $t_{T}$ (**b**) for both resonance modes. Shaded regions correspond to the critical coupling regime that occurs at $t_{S-\mathrm{critical}}$ (**a**) and $t_{T-\mathrm{critical}}$ (**b**), resulting in perfect absorption for both resonances. Above $t_{T-\mathrm{critical}}$ the scattering on the nanodiscs become dominant and absorption decreases. Below and above $t_{S-\mathrm{critical}}$ the coupling weakens, thus the absorption of the system is reduced.

The thickness of the dielectric spacer within the MIM configuration is an important parameter to achieve perfect absorption (A≈1) and narrowband resonances (FWHM below 15 nm) [45]. The prior studies explained that the perfect absorption can be achieved periodically at various dielectric thicknesses over a frequency range [33,34]. The dielectric thickness is also related to the refractive index of the material. Figure 4a presents the simulated absorption peak amplitude for both resonance modes as a function of $t_{S}$ when the MgF${}_{2}$ spacer thickness is varied between 20–70 nm at 10 nm steps. Dielectric thickness is critically important in achieving the perfect absorption as it controls the coupling strength between plasmons excited at the top nanodisc and the bottom gold film. As $t_{S}$ changes, the absorption amplitudes of both Peak1 and Peak2 change. As our goal is to achieve maximum absorption for both peaks, the critical thickness is obtained at $t_{S}=40$ nm, where the radiative and intrinsic damping rates are equal ($\gamma_{r}\approx \gamma_{i}$), and the reflection is minimized, justifying the critical coupling. As the dielectric thickness increases before the $t_{S-\mathrm{critical}}$, the near-field coupling between the bottom gold film and the nanodisc plasmons strengthen. At the critical thickness these plasmons strongly couple yielding maximum absorption. Increasing the thickness above $t_{S-\mathrm{critical}}$ weakens the coupling and thus reducing the absorption of the system. A similar study is applied to the nanodisc thickness $t_{T}$, shown at Figure 4b. The dependence of the absorption on $t_{T}$ and $t_{S}$ are very similar. At a critical thickness (t${}_{T-\mathrm{critical}}=40$ nm) the absorption is maximized for both Peak1 and Peak2. Below the critical thickness the plasmon coupling strength between the gold layers weakens resulting in low absorption. Above the critical thickness, the scattering on the nanodisc becomes dominant and absorption decreases. When the same nanodisc antenna design is placed on only MgF${}_{2}$ layer or on only Au layer, perfect absorption cannot be observed (Figure S3).

**Figure 5:**
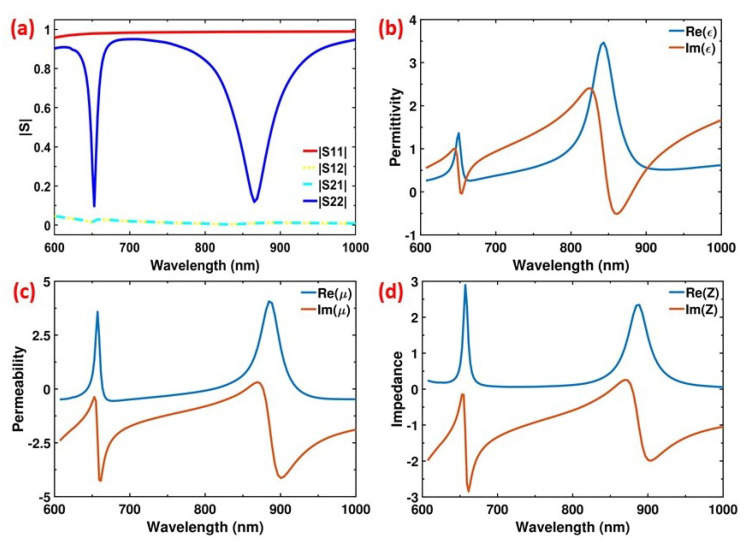
(**a**) Magnitude of the simulated complex S-parameters of the proposed PA for both forward and backward propagating source directions. Effective optical parameters, permittivity (**b**), permeability (**c**), and impedance (**d**) are extracted from the S-parameters as functions of the wavelength. At both resonant wavelengths, the real part of the effective impedance of the proposed PA satisfies the impedance matching condition Re(Z1)≈Re(Z2)≈Zair/vacuum≈1.

In earlier reports of metamaterial PAs perfect absorption over a narrow frequency range is explained by minimizing the reflection with perfect impedance matching $\left(z=\sqrt{\mu /\epsilon }=1\right)$ [32]. The antenna impedance usually depends upon the geometrical parameters of the system as well as the physical properties of the material used to compose the device. To calculate the impedance of the nanodisc antennas at the resonant frequencies, we consider the whole system as an effective medium with effective optical properties, i.e., permittivity $\epsilon_{\mathrm{eff}}$ and permeability $\mu_{\mathrm{eff}}$. FDTD simulations are performed to retrieve complex scattering matrix elements (S-parameters) using the S-parameter analysis group of the software. Then, effective permittivity, permeability, and impedance are extracted from the S-parameters as discussed in Supplementary Materials [40,46]. Our proposed system depends strongly on the propagation direction due to the bottom metallic plate which block the transmission through the PA, thus we performed two simulations: one for the forward (+z direction) and one for the backward (−z direction) propagating source directions. Figure 5a represents the simulated S-parameter amplitudes for both propagating directions. $S_{11}$ and $S_{21}$ correspond to the reflection and transmission coefficients for the backward propagating source, respectively; $S_{22}$ and $S_{12}$ correspond to the reflection and transmission coefficients for the forward propagating source, respectively. When the source is propagating along the −z direction, almost all the light gets reflected ($S_{11}\approx 1$) (Figure 5a) as its thickness is more than the skin depth, thus no light can even reach the dielectric spacer and the nanodiscs which are responsible for the resonating behavior. On the other hand, in the case for forward propagation (+z), a resonating behavior is observed due to the appearance of two dips in the reflection coefficient $S_{22}$ at the relevant resonance frequencies (Figure 5a). Meanwhile, for either case transmission is forbidden as transmission coefficients are almost negligible ($S_{21}\approx S_{12}\approx 0$). Figure 5b–d shows the effective permittivity, permeability, and impedance of the PA. At Peak1, permittivity and permeability are calculated as $\epsilon_{R1}\approx 1.04-0.02i$ and $\mu_{R1}\approx 1.34-0.37i$, leading to the effective impedance $Z_{1}\approx 1.15-0.14i$. Similarly, for Peak2 the parameters are calculated as $\epsilon_{R2}\approx 1.58-0.46i$, $\mu_{R2}\approx 1.48+0.26i$, and $Z_{2}\approx 0.93-0.21i$. Given that $Z_{\mathrm{air}}\approx 1$, the real parts of the effective impedances at Peak1 (1.15) and Peak2 (0.93) closely satisfy the condition of impedance matching with the free space. Hence, the reflection is minimum at the mentioned peaks, and the perfect absorption is achieved at both resonant frequencies.

**Figure 6:**
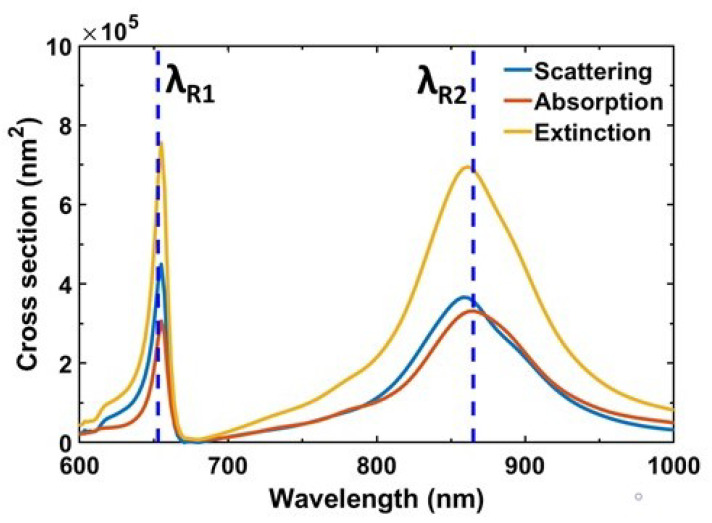
Simulated scattering, absorption and extinction (scattering + absorption) cross sections as a function of wavelength for the PA with optimized geometrical parameters. The vertical blue dashed lines identify the resonance wavelengths ($\lambda_{R1}\approx 653$ nm, $\lambda_{R2}\approx 865$ nm). At both resonances, scattering and absorption cross sections are nearly identical, which justifies the critical coupling and minimum reflection.

As the PA system contains nanodisc antennas, it is crucial to study their individual scattering properties to minimize the total reflection arising from the system. Towards this aim, we calculate the absorption ($C_{\mathrm{abs}}$), scattering ($C_{\mathrm{sca}}$) and extinction ($C_{\mathrm{ext}}$) cross sections using FDTD simulations. A total-field scattered-field (TFSF) source is used to illuminate the single unit cell, and scattering and absorption cross sections are measured utilizing the cross section analysis group of the software. Figure 6 shows that at both Peak1 and Peak2 ($\lambda_{R1}$, $\lambda_{R2}$) absorption cross section is almost identical to the scattering cross section, and also equal to half of the extinction cross section, i.e., $C_{\mathrm{ext}}\approx 2C_{\mathrm{sca}}\approx 2C_{\mathrm{abs}}$. This relation occurs whenever the system is in the critical coupling regime such that the radiative and intrinsic damping rates become identical ($\gamma_{r}\approx \gamma_{i}$), yielding near-unity absorption [34]. Moreover, it is worth mentioning that at the critical coupling regime absorption and scattering cross sections can be much greater than the geometrical cross section $C_{\mathrm{geo}}=\pi D^{2}/4$ of the nanoantenna. In this study, we find that the absorption and scattering cross sections are almost 10 times bigger than the geometrical cross section of the nanodisc antenna.

## 3. A Potential Application: Biosensing

**Figure 7:**
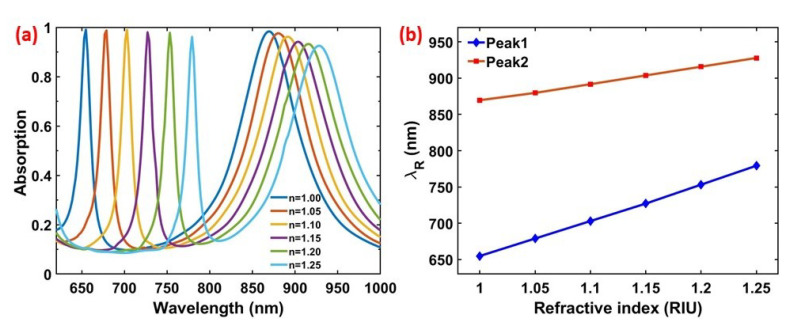
(**a**) Simulated absorption spectra of the PA within different refractive index backgrounds; a 0.05 RIU change delivers a significant traceable red shift. (**b**) The relation between Peak1 and Peak2 resonance wavelengths ($\lambda_{R}$) with the surrounding refractive index. The sensitivity (slope) for both peaks are calculated as S${}_{1}\approx$ 500 nm/RIU and S${}_{2}\approx$ 235 nm/RIU.

The plasmonic resonance behavior of the MIM design strongly depends on the refractive index of the surrounding environment. The strong near-field enhancements explained in Figure 2a,b intensify the interactions between incident electromagnetic radiation and nanodiscs, thus any small refractive index change around the nanodisc causes a spectral shift on the resonance. Having narrow resonances significantly improves tracing the resonance shift and makes the narrow-band resonances an ideal instrument for refractive index-based sensing. In order to analyze the sensing capabilities of the designed PA, a variety of surroundings with different refractive indices are tested theoretically. Figure 7a shows the resonance behaviour of the optimized PA within different refractive index environments. A red shift is observed for both Peak1 and Peak2 as the surrounding refractive index increase from 1 to 1.25 with 0.05 refractive index unit (RIU) steps. Figure 7b shows the relation between the resonance wavelength and the environmental refractive index. The refractive index sensitivity ($S=▵\lambda_{R}/▵n$) for resonance modes are calculated as $S_{1}\approx 500$ nm/RIU and $S_{2}\approx 235$ nm/RIU, respectively. Furthermore, the quality factor ($Q=\lambda_{R}/\mathrm{FWHM}$) for the resonant peaks are calculated as $Q_{1}\approx 44$ and $Q_{2}\approx 11$, respectively. Figure 7 reveals that Peak1 is more sensitive to the changes in the surrounding. This result is expected as the near-field enhancement for the first resonance mode occurs on the top edges of the nanodiscs and it is more exposed to the surrounding compared to the second resonance where the near-field is enhanced on the lower edges of the nanodiscs. The sensitivity of 500 nm/RIU can enable detection of analytes at very low concentrations [47], thus the presented PA is an ideal candidate for the sensing applications in the designed frequency regime.

## 4. Conclusions

We numerically investigate and present a narrow-band multi-resonant plasmonic perfect absorber based on a multilayer MIM configuration operating within the wavelength range of 600 to 950 nm. The FWHM of the optimized PA could go down to 15 nm corresponding to a resonance quality factor Q=44. Our multi-resonant PA features PSP and LSP excitations at distinct frequencies, in contrast to single-resonant PAs. The LSP and PSP nature of the plasmonic resonances are verified with near-field analysis and the geometric dependence. MgF${}_{2}$ and the antenna thicknesses effect the perfect absorption directly. Moreover, the physical origin of the perfect absorption on the presented PA is explained in terms of both impedance matching and critical coupling phenomena. The real part of the effective impedance of the PA is calculated as $Z_{1}\approx 1.15$ for Peak1 and $Z_{2}\approx 0.93$ for Peak2, closely matching the free space impedance. The calculated absorption cross section of the PA is almost identical to the scattering cross section for both Peak1 and Peak2 and equal to the half of the extinction cross section ($C_{\mathrm{ext}}\approx 2C_{\mathrm{sca}}\approx 2C_{\mathrm{abs}}$) confirming the critical coupling condition. Thus, the radiative and intrinsic damping rates become identical ($\gamma_{r}\approx \gamma_{i}$), which explains the zero reflection and the perfect absorption of the presented PA. Finally, biosensing capacity of the system is quantified as a potential application. The refractive index sensitivity of the PA could go as high as 500 nm/RIU. We believe that its unique optical features makes the presented multi-resonant PA a favorable platform for biosensing and multi-resonant contrast agent based bioimaging.

## Supplementary Materials

The following are available online at https://www.mdpi.com/1996-1944/13/22/5140/s1. Figure S1: Effect of the polarization angle, Figure S2: Effect of the incidence angle, Figure S3: The comparison against single antenna PA, Figure S4: The effect of dielectric spacer material, Figure S5: The effect of antenna shape on the perfect absorption, Figure S6: Calculation of the effective material properties.
