# Supplementary material for: A Narrow-Band Multi-Resonant Metamaterial in Near-IR
Source: Materials (Basel). 2020 Nov 14;13(22):5140. doi: 10.3390/ma13225140 (PMC7696973; doi:10.3390/ma13225140)
Supplement: Supplementary file 1 [file materials-13-05140-s001.pdf]

materials

Supplementary File

A Narrow-Band Multi-Resonant Metamaterial in Near-IR

Farhan Ali [1,2] and Serap Aksu [1,*]

[1] Department of Physics, Bilkent University, Ankara 06800, Turkey; fali19@ku.edu.tr
[2] Department of Physics, Koc University, Istanbul 34450, Turkey
* Correspondence: saksu@ku.edu.tr

Received: 28 September 2020; Accepted: 26 October 2020; Published: 14 November 2020

1. Effect of the Polarization Angle

In order to investigate the effect of polarization angle (ϕ) of the incident light source, we illuminate the perfect absorber with optimized structural parameters (P = 600 nm, D = 180 nm, t_B = 100 nm, t_S = t_T = 40 nm) using different polarization angles (ϕ = 0°, 45°, 90°). The source is normally incident ($\theta = 0$). Figure S1 shows the absorption spectrum as a function of wavelength for three different polarization states. Due to the circular symmetry of the top nanodisks array, there is no significant change on the absorption.

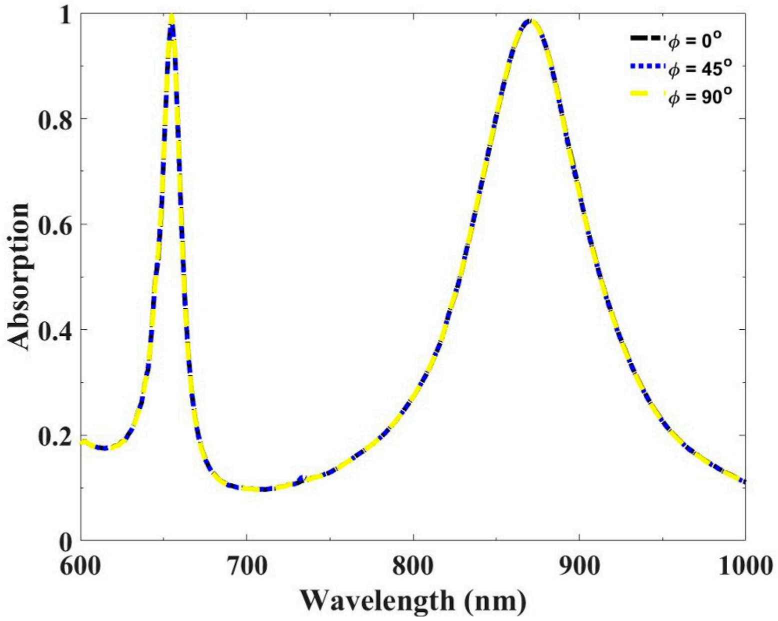

Figure S1. Absorption spectra of the optimized perfect absorber for three different polarization states of the incident light. The perfect absorber is polarization insensitive.

2. Effect of the Incidence Angle

To observe the dependence of the incidence angle (θ), we illuminate the perfect absorber with optimized structural parameters (P = 600 nm, D = 180 nm, t_B = 100 nm, t_S = t_T = 40 nm) by an x-polarized light source at different incidence angles. Figure S2 shows the absorption spectrum as a function of wavelength when the incident angle changes from 0–25° with a step of 5°. When the angle is introduced to the incident light the resonance bandwidth changes dramatically. As the angles increase, both Peak1 and Peak2 shows broader bandwidths at the same resonance wavelengths. Angle changes up to 10° does not significantly affect the quality of the perfect absorption.

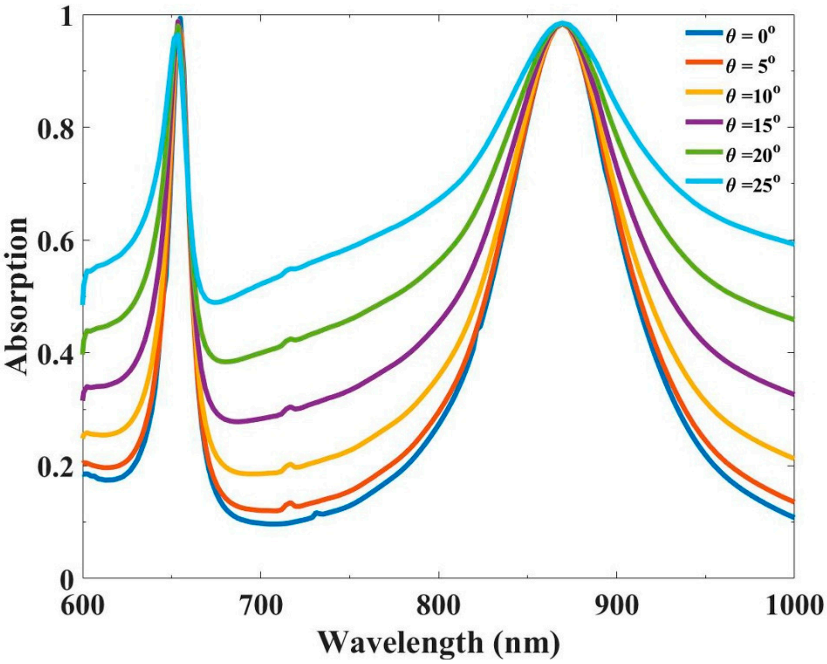

Figure S2. Absorption spectra of the optimized perfect absorber for different incidence angles (θ). Increasing θ results in the broadening of the resonance bandwidths of both the peaks. Angle changes up to 10° does not significantly affect the quality of the perfect absorption.

3. The Comparison against Single Antenna PA

The need for three layered metal-insulator-metal (Au-MgF_2-Au) configuration is previously presented in several articles [1]. Here, to emphasize the need for the three-layer system, we compare the optical response of single antenna (Au nanodisc antenna on MgF_2) and (Au nanodisc antenna on Au bottom layer) in Figure S3. When the nanodisc antenna design is placed only on a MgF_2 substrate, we observe very broad transmission resonance and very low absorption intensity. Similarly, when the same top antenna design is placed on a Au substrate, we observe a very low absorption intensity. The theoretical and experimental [1] results clearly show that the PA can exhibit higher absorption intensity when a dielectric spacer with finite thickness is sandwiched between ground metal plate and top metal antenna layer.

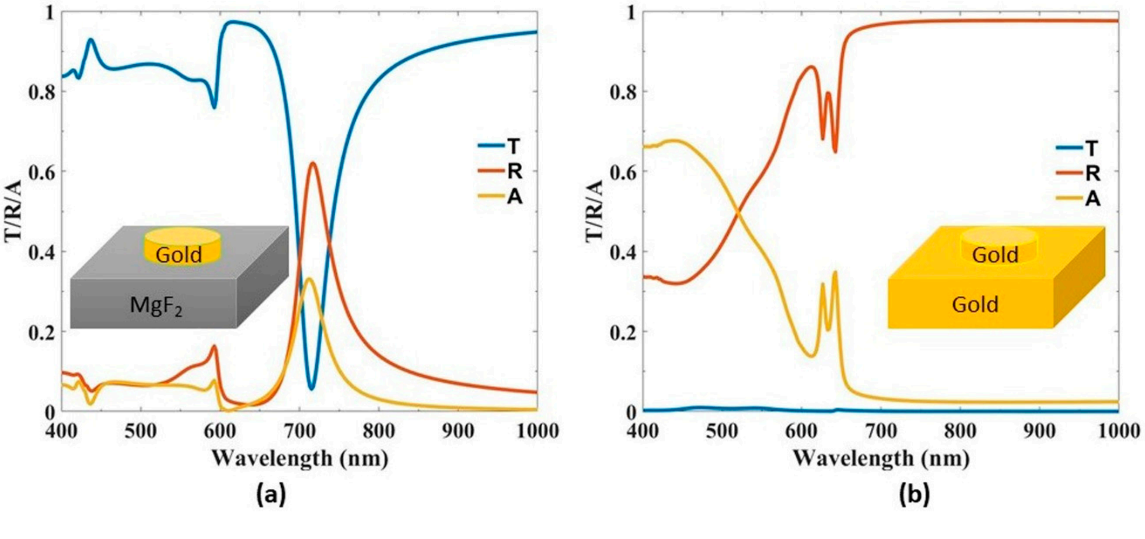

Figure S3. Absorption spectra of the optimized nanodisc antenna array (**a**) on MgF_2 only; (**b**) on gold only. None of the configurations above provide perfect absorption.

4. The Effect of Dielectric Spacer Material

The effect of dielectric spacer material for perfect absorption is discussed in Figure S4. As the refractive index of the spacer changes the spacer thickness has be tuned to achieve perfect absorption. Figure S4 shows the evolution of absorption intensity as a function of refractive index (n_s) for the proposed absorber with optimum parameters. When n_s changes between 1 and 1.7 the absorption is always higher than 90% for the same spacer thickness. With a critical thickness tuning perfect absorption can be obtained at all refractive indices. Figure S4b shows that resonant wavelength of Peak2 is more sensitive to the refractive index change. Resonant wavelength for both Peak1 and Peak2 depend linearly on the n_s in the studied frequency range.

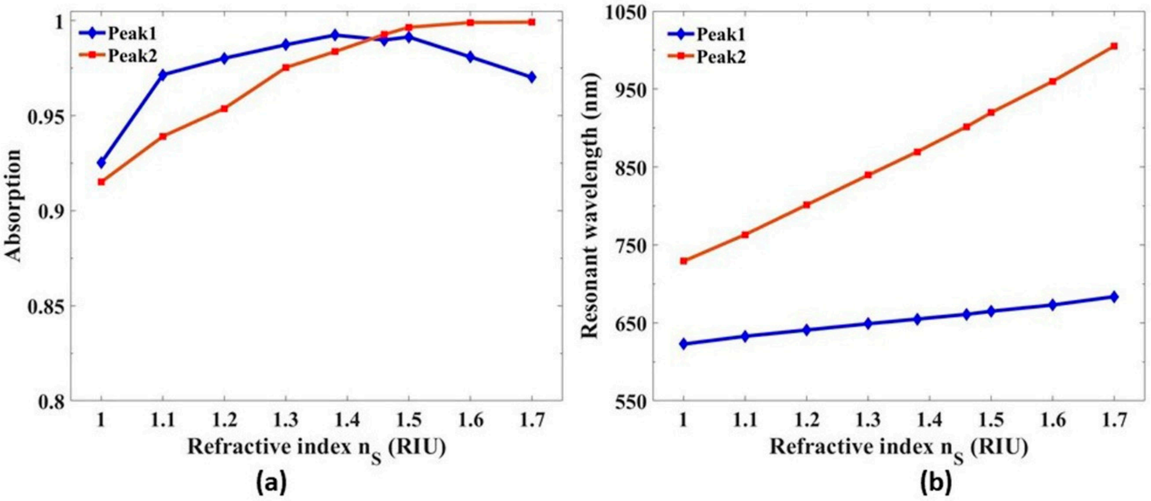

Figure S4. (**a**) The effect of dielectric spacer refractive index on absorption intensity for the same thickness. As the refractive index of the spacer changes between 1–1.7, the absorption is always higher than 90%; (**b**) The effect of spacer refractive index on resonance wavelengths. Peak2 is more sensitive to refractive index change, as it is a LSP resonance.

5. The Effect of Antenna Shape on the Perfect Absorption

The effect of the nanoantenna shape on the absorption and resonance wavelength is investigated using six different shapes. Figure S5b shows the absorption spectra for the shapes given in Figure S5a. The perfect absorption (>90%) is achieved with other shapes as well. The absorption intensity for all the systems is above 90% and very similar to each other. Figure S5c shows the resonant wavelength as a function of shape. Change of nanoantenna shape affects only the second resonance (Peak2). This result is expected as Peak2 is a LSP resonance that depends on the individual antenna properties.

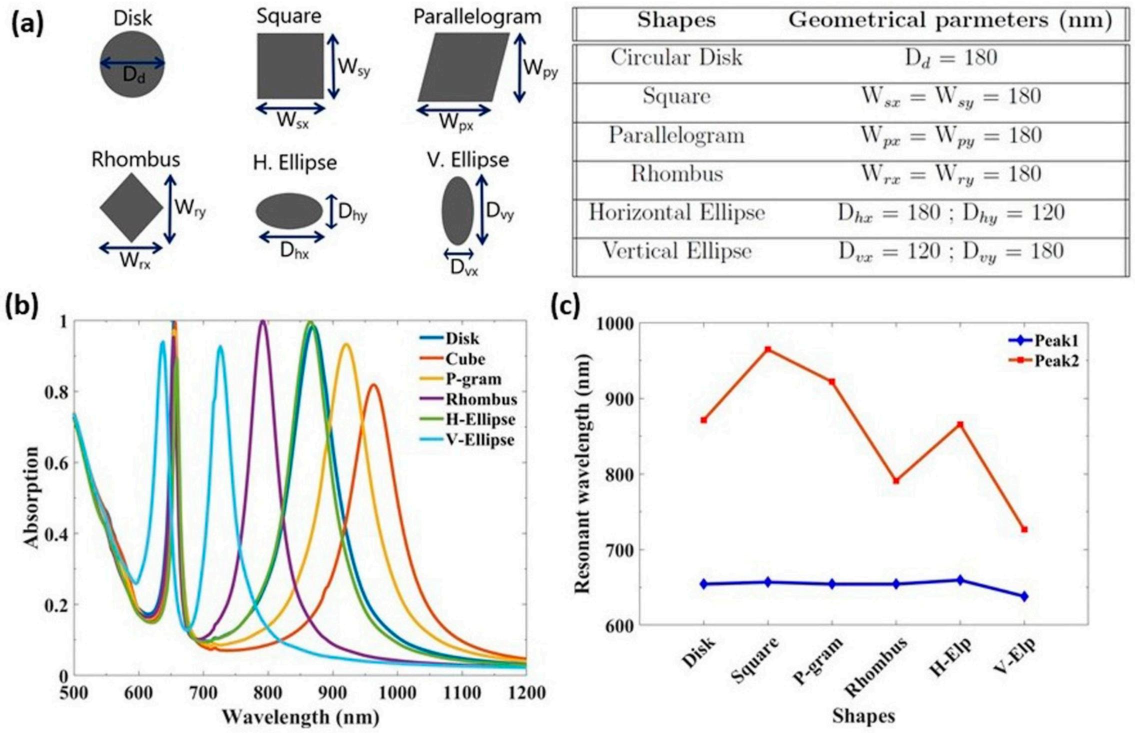

Figure S5. The effect of different shapes shown on: (**a**) the absorption; (**b**) and resonant wavelength; (**c**) The absorption intensity for all the systems is above 90% and very similar to each other.

6. Calculation of the Effective Material Properties

To explain the physical mechanism of the perfect absorption of the proposed perfect absorber, we calculate the impedance of the system at the resonant frequencies. An effective medium approach is utilized where the whole system is considered as a single effective medium with effective material properties (ε_{eff}, μ_{eff}). We first perform the simulations to obtain S-parameters related to the system, which then used to retrieve the effective material properties. S-Parameters are the complex amplitude reflection and transmission coefficients of the system and are calculated from the built-in S-Parameters analysis group in the Lumerical FDTD solutions. Figure S6 shows a schematic explaining the principle to calculate these complex reflection / transmission coefficients. Only unit cell of the proposed metamaterial absorber is considered and illuminated by a plane wave source from both the direction (inputs I_1 and I_2), then the output signal O_1 and O_2 are given by:

$$\begin{pmatrix} O_1 \\ O_2 \end{pmatrix} = \begin{pmatrix} S_{11} & S_{12} \\ S_{21} & S_{22} \end{pmatrix} \begin{pmatrix} I_1 \\ I_2 \end{pmatrix} \tag{1}$$

where, S_{11}, S_{22} are the complex reflection, and S_{21}, S_{12} are the complex transmission coefficients for the I_1 and I_2 incidence signals, respectively. These parameters can be defined from the incident (E_i) and reflected (E_r)/transmitted (E_t) electric fields as $S_{11} = (E_r)/(E_i)$ and $S_{21} = (E_t)/(E_i)$ for I_1 incident plane wave and vice versa for the I_2 incidence.

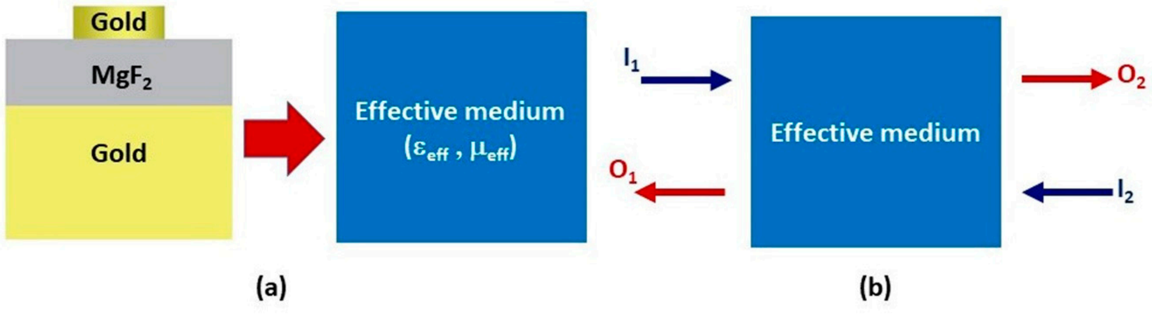

Figure S6. (**a**) A schematic of the perfect absorber's unit cell (cross-sectional view) as an effective medium with effective material properties (ε_{eff}, μ_{eff}); (**b**) A schematics for S-parameter calculation from an effective medium when excited with a light source from both directions (I_1: forward propagation, I_2: backward propagation) with corresponding output signals O_1 and O_2.

Once we have the S-parameters, we can retrieve the impedance (Z) and other effective properties of the device by following equations [2]:

$$Z_{eff} = \frac{(T_{22} - T_{11}) \pm \sqrt{(T_{22} - T_{11})^2 + 4T_{12}T_{21}}}{2T_{21}} \tag{2}$$

$$n_{eff} = \frac{1}{kd}\cos^{-1}\left(\frac{1 - S_{11}S_{22} + {S_{21}}^2}{2S_{11}}\right) \tag{3}$$

$$\varepsilon_{eff} = \frac{n_{eff}}{Z_{eff}} \tag{4}$$

$$\mu_{eff} = n_{eff}\, z_{eff} \tag{5}$$

where, T_{ij} are transfer matrix elements given as:

$$T_{11} = \frac{(1 + S_{11})(1 - S_{22}) + S_{21}S_{12}}{2S_{21}} \tag{6}$$

$$T_{12} = \frac{(1 + S_{11})(1 + S_{22}) - S_{21}S_{12}}{2S_{21}} \tag{7}$$

$$T_{21} = \frac{(1 - S_{11})(1 - S_{22}) - S_{21}S_{12}}{2S_{21}} \tag{8}$$

$$T_{22} = \frac{(1 - S_{11})(1 + S_{22}) + S_{21}S_{12}}{2S_{21}} \tag{9}$$

References:

1. Korkmaz, S.; Turkmen, M.; Aksu, S. Mid-infrared narrow band plasmonic perfect absorber for vibrational spectroscopy. *Sens. Act. A: Phys.* **2020**, *301*, 111757.
2. Smith, D.R.; Vier, D.C.; Koschny, T.; Soukoulis, C.M. Electromagnetic parameter retrieval from inhomogeneous metamaterials. *Phys. Rev. E.* **2005**, *71*, 036617.

Publisher's Note: MDPI stays neutral with regard to jurisdictional claims in published maps and institutional affiliations.

© 2020 by the authors. Licensee MDPI, Basel, Switzerland. This article is an open access article distributed under the terms and conditions of the Creative Commons Attribution (CC BY) license (http://creativecommons.org/licenses/by/4.0/).
